# GLI3 Links Environmental Arsenic Exposure and Human Fetal Growth

**DOI:** 10.1016/j.ebiom.2015.04.019

**Published:** 2015-05-01

**Authors:** Emily F. Winterbottom, Dennis L. Fei, Devin C. Koestler, Camilla Giambelli, Eric Wika, Anthony J. Capobianco, Ethan Lee, Carmen J. Marsit, Margaret R. Karagas, David J. Robbins

**Affiliations:** a Molecular Oncology Program, DeWitt Daughtry Family Department of Surgery, University of Miami Miller School of Medicine, Miami, FL 33136, USA; b Department of Pharmacology and Toxicology, Geisel School of Medicine at Dartmouth, Hanover, NH 03755, USA; c Department of Biostatistics, University of Kansas Medical Center, Kansas City, KS 66160, USA; d Department of Epidemiology, Geisel School of Medicine at Dartmouth, Hanover, NH 03755, USA; e Department of Biochemistry and Molecular Biology, University of Miami Miller School of Medicine, Miami, FL 33136, USA; f Molecular Oncology Program, Sylvester Comprehensive Cancer Center, University of Miami Miller School of Medicine, Miami, FL 33136, USA; g Department of Cell and Developmental Biology, Vanderbilt University Medical Center, Nashville, TN 37232, USA

**Keywords:** CI, confidence interval, IQR, interquartile range, MCL, maximum contaminant level, As, arsenic, U-As, total urinary arsenic concentration, HH, HEDGEHOG, Arsenic, Development, GLI3, Placenta, Sex, Birth Weight

## Abstract

Although considerable evidence suggests that in utero arsenic exposure affects children's health, these data are mainly from areas of the world where groundwater arsenic levels far exceed the World Health Organization limit of 10 μg/L. We, and others, have found that more common levels of in utero arsenic exposure may also impact children's health. However, the underlying molecular mechanisms are poorly understood. To address this issue, we analyzed the expression of key developmental genes in fetal placenta in a birth cohort of women using unregulated water supplies in a US region with elevated groundwater arsenic. We identified several genes whose expression associated with maternal arsenic exposure in a fetal sex-specific manner. In particular, expression of the HEDGEHOG pathway component, *GLI3*, in female placentae was both negatively associated with arsenic exposure and positively associated with infant birth weight. This suggests that modulation of *GLI3* in the fetal placenta, and perhaps in other fetal tissues, contributes to arsenic's detrimental effects on fetal growth. We showed previously that arsenic-exposed NIH3T3 cells have reduced GLI3 repressor protein. Together, these studies identify GLI3 as a key signaling node that is affected by arsenic, mediating a subset of its effects on developmental signaling and fetal health.

## Highlights

• In utero arsenic exposure associates with the expression of several key developmental genes in the fetal placenta.
• There is extensive sexual dimorphism in the associations between placental gene expression and in utero arsenic exposure.
• GLI3 expression in the female fetal placenta associates with arsenic exposure and may mediate its effects on fetal growth.

## 1 Introduction

Arsenic is a well-known toxic metalloid, and both a naturally occurring and anthropogenic environmental contaminant (Hughes et al., 2011). As such, arsenic levels are regulated in public drinking water supplies in the US and other countries. In accordance with World Health Organization guidelines, the maximum contaminant level (MCL) for arsenic set by the US Environmental Protection Agency (EPA) is 10 μg/L. However, in some parts of the world, arsenic contamination of drinking water can reach several thousand micrograms per liter (Nordstrom, 2002). Ingestion of such highly contaminated water by pregnant women, leading to in utero arsenic exposure of the developing fetus, is associated with several adverse birth conditions, including low birth weight (Huyck et al., 2007, Llanos and Ronco, 2009, Rahman et al., 2009, Xu et al., 2011), increased risk of infection (Rahman et al., 2011), and spontaneous abortion and infant mortality (Myers et al., 2010, Sohel et al., 2010), as well as increased risk of certain diseases in later life (reviewed in (Farzan et al., 2013)). Less is understood about lower exposure levels, despite their relevance to millions of people worldwide (Nordstrom, 2002). Additionally, while there is evidence to suggest that the response to in utero arsenic exposure varies by fetal sex (Hamadani et al., 2011, Raqib et al., 2009, Xu et al., 2011), the molecular basis for such differences is unknown. Therefore, there is an urgent need to characterize the effects of in utero arsenic at common exposure levels, in a manner that accounts for fetal sex, in order to prevent and treat adverse health outcomes in at-risk populations. With this aim in mind, the current study was performed on a cohort of pregnant women who were exposed to arsenic in drinking water at levels that span above and below the current US MCL.

Embryonic development is a highly regulated process that involves numerous biological mechanisms, many of which are controlled by three major developmental signaling pathways: HEDGEHOG (HH), NOTCH and WNT (Clevers, 2006, Briscoe and Therond, 2013, Hori et al., 2013). Cells receiving these signals undergo proliferation, differentiation, and apoptosis, allowing the formation of sophisticated structures and patterns. Activation of each of these signal transduction cascades leads to the transcriptional regulation of a particular set of target genes. These three signaling pathways are also involved in the self-renewal of tissue-specific stem cells that drive organ development and maintain tissue homeostasis in the adult. These stem cells often exhibit high-level expression of “stemness” genes (Boiani and Scholer, 2005). During development, there are extensive cooperative and antagonistic interactions between these pathways, which ultimately determine cell fate.

In keeping with their roles in cellular differentiation and embryogenesis, mutations in components of the HH, NOTCH or WNT pathways lead to human developmental disorders. For example, holoprosencephaly (HPE), a birth defect that affects the formation of midline structures and causes facial defects including cleft lip, often results from loss-of-function mutations in one allele of the HH family member SONIC HEDGEHOG (SHH) (Belloni et al., 1996, Roessler et al., 1996). This example underscores the susceptibility of humans to alterations in the activity of these signaling pathways. Although severe disruptions of these developmental pathways in humans likely lead to spontaneous abortion, stillbirth, or distinct birth defects, more subtle alterations in pathway activity, such as those resulting from environmental exposures, may also perturb fetal development and impact long-term health.

In this study of a US pregnancy cohort, we sought to identify pivotal developmental genes whose expression in fetal placenta associated with common levels of arsenic exposure. The placenta is a specialized organ that is critical for normal fetal development and survival. In addition, the fetal portion of the placenta shares its genotype and environment with the fetus proper, and so may be viewed as a sentinel for the fetus that can be analyzed in a non-invasive manner. We also examined the association of our candidate developmental genes with infant birth weight, the outcome of fetal growth, which is one important aspect of fetal development. We report here that the placental expression of several key developmental genes associates with in utero arsenic exposure, and further, that many of these genes associate with arsenic in a fetal sex-specific manner. Moreover, we found that expression of the HH pathway gene *GLI3* was negatively associated with arsenic exposure but positively associated with birth weight among females. This observation suggests a model whereby in utero arsenic exposure of the female fetus results in reduced placental and possibly fetal *GLI3* expression, which in turn leads to reduced birth weight.

## 2 Materials and Methods

### 2.1 Study Design

The study cohort consisted of 133 pregnant women enrolled in the ongoing New Hampshire Birth Cohort Study (NHBCS), which began in January 2009 (Gilbert-Diamond et al., 2011). Eligibility criteria have been described previously (Koestler et al., 2013). Briefly, subjects were English-speaking, literate, mentally competent women of 18–45 years of age with a singleton pregnancy, whose home water supply was from a private well, and who had not changed residence since their last menstrual period. Participants provided written informed consent before enrolling in the study. Demographic data, pregnancy history and outcome, and lifestyle factor information were collected from questionnaires and review of prenatal and delivery records. All research involving human participants has been approved by The Committee for the Protection of Human Subjects at Dartmouth College.

### 2.2 Sample Collection and Arsenic Measurement

Details of urine and household water sample collection and arsenic measurement have been described previously (Koestler et al., 2013). Briefly, spot urine samples were collected at approximately 24–28 weeks gestation and analyzed for individual arsenic species at the University of Arizona using a high-performance liquid chromatography inductively coupled plasma mass spectrometry (ICP-MS) system, with a detection limit ranging from 0.10 to 0.15 μg/L for the individual arsenic species. Measurements below the limit of detection were recorded as the median value between 0 μg/L and the detection limit for that arsenic species. Total urinary arsenic concentration, U–As, was calculated by summing the inorganic arsenic (iAs) species, arsenite (As^III^) and arsenate (As^V^), plus dimethylarsinic acid (DMA^V^) and monomethylarsonic acid (MMA^V^), and excluding arsenobetaine. Household water samples were collected by the study participants using EPA-approved I-Chem containers (VWR), and total arsenic concentration was analyzed by ICP-MS at the Trace Element Analysis Core at Dartmouth. The detection limit ranged from 0.009 to 0.074 μg/L.

### 2.3 Placenta Biopsy and Gene Profiling

Placenta samples were obtained at the time of delivery. Biopsies were taken at the base of the umbilical cord insertion, measuring roughly 1 cm deep to ensure the tissue was of fetal origin, and 1–2 cm across, and were immersed immediately in RNAlater (Life Technologies) and placed at — 80 °C for long-term storage. RNA extraction and subsequent gene profiling were performed in three batches (44, 44 and 45 samples respectively). Roughly 200 mg of placenta was homogenized in 1 ml Tri Reagent (Molecular Research Center) using a motorized homogenizer, and further purified using RNeasy mini columns (Qiagen). 100 ng of high-quality RNA from each sample was subjected to gene expression analysis using the Nanostring system (Nanostring Technologies) at the Oncogenomics Core Facility of the University of Miami. The Nanostring codeset was custom-designed for 29 development-related genes, and 5 housekeeping genes (*ATCB*, *GAPDH*, *HPRT1*, *RPL19*, and *RPLP0*) (Table 1). Two distinct probes were designed for *GLI1* and *PTCH1* (GLI1/GLI1-2, PTCH1/PTCH1-2). Raw count data was first normalized to the spike-in positive and negative controls to account for assay efficiency and then normalized to the geometric mean of the expression of the 5 housekeeping genes using nSolver software (Nanostring Technologies).

### 2.4 Statistical Analysis

Gene expression data was first batch-adjusted using the COMBAT method (Johnson et al., 2007), which uses a location and scale adjustment to standardize the mean and variability in expression levels across batches. Principal component analysis was then performed on the batch-adjusted data to ensure that batch effects had been successfully attenuated. Using the batch-adjusted data, a series of multivariable linear regression models were used to examine the association between U–As and gene expression for each of the candidate genes, as described previously (Fei et al., 2013). Multivariable regression models were fitted to the cohort as a whole and after stratification by infant sex. We modeled log-transformed gene expression as a function of log-transformed U–As and adjusted for maternal age at delivery. This adjustment was based on analyses using a series of linear regression models to identify those covariates that associated with arsenic exposure or birth weight, among potential confounders, i.e., maternal age, maternal smoking status (never, former, current), maternal education level, infant birth weight, infant sex and gestational age. Unadjusted versions of all linear regression analyses are provided in Table S4. As shown previously (Fei et al., 2013), a power analysis indicated adequate statistical power for detecting low-moderate correlations between U–As levels and gene expression (absolute correlation (r) = 0.24; power = 80%) at a significance level of 0.05 and a study sample size of 133 subjects. All aforementioned analyses were carried out using the R statistical program, version 2.13 (http://cran.r-project.org/). Similar methods were used to examine the associations of candidate gene expression with infant birth weight, adjusting for maternal age and gestational age, which were identified as confounders as described above.

### 2.5 Funding Sources

This publication was made possible by U.S. Environmental Protection Agency (US EPA) grant RD-83544201, National Institute of Environmental Health Sciences (NIEHS) grant P01 ES022832, a CTSA grant from NCATS awarded to the University of Kansas Medical Center for Frontiers: The Heartland Institute for Clinical and Translational Research # KL2TR000119 (DCK), and NIH grants R01GM081635 and R01GM103926 (EL). Its contents are solely the responsibility of the grantee and do not necessarily represent the official views of the US EPA. Further, the US EPA does not endorse the purchase of any commercial products or services mentioned in the publication. The funding bodies did not play any role in the writing of the manuscript or the decision to submit it for publication.

## 3 Results

### 3.1 Characteristics of the Study Cohort

The study cohort consisted of 133 mother–child pairs enrolled from prenatal clinics in the state of New Hampshire, USA (Table 1). The average age at the time of enrollment was 31.1 years and the average gestational period was 39.5 weeks. Only a small percentage reported smoking during pregnancy (5/133, 3.8%). Average pre-pregnancy body mass index (BMI) was 24.9 kg/m^2^ and parity was 1.1. About half the infants were male, and the average infant birth weight was 3.4 kg. The median arsenic concentration in household tap water was 0.36 μg/L (interquartile range [IQR] 0.02–3.55) and 16% of participants (21/133) consumed drinking water containing arsenic at concentrations above the current MCL of 10 μg/L. The median concentration of total urinary arsenic (U–As), which includes all arsenic species except arsenobetaine, was 4.4 μg/L (IQR 1.8–11.9). We found a strong association between the participants' household water arsenic concentrations and urinary arsenic concentrations, excluding arsenobetaine (Pearson correlation = 0.55, p-value < 0.001).

### 3.2 Expression of GLI1 and POU5F1 in the Fetal Placenta is Associated with Maternal Arsenic Exposure

**Fig. 1:**
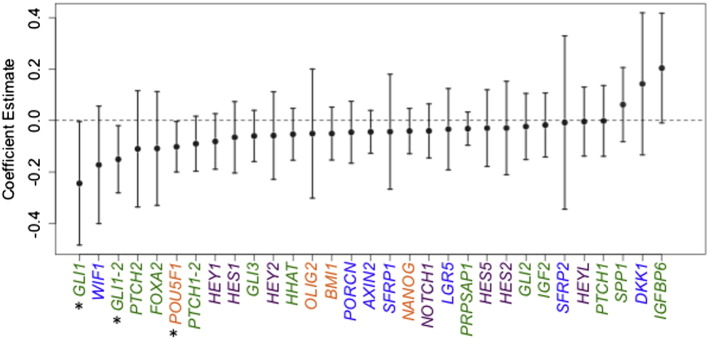
Associations between placental gene expression and maternal U–As. Multivariable linear regression analyses were performed to determine the association between maternal U–As and placental gene expression. The analyses were adjusted for maternal age. GLI1/GLI1-2 and PTCH1/PTCH1-2 are sequence-distinct Nanostring probes designed to measure the expression of *GLI1* and *PTCH1*. Dots depict coefficient estimates and error bars represent 95% CIs. Significant associations are those with 95% CIs not crossing zero (dotted line) and are marked by asterisks (*P < 0.05). Green; HH pathway-related genes, purple; NOTCH pathway-related genes, blue; WNT pathway-related genes, orange; stemness genes.

We used maternal U–As as an estimate of arsenic exposure of the fetus because it provides a reliable indication of arsenic concentration in blood (Marchiset-Ferlay et al., 2012), and arsenic is known to readily cross the placenta (Concha et al., 1998). We measured the expression of candidate genes in the fetal portion of the placenta to examine how arsenic exposure might affect key developmental genes and signaling pathways during fetal development. The gene list comprises key components and consensus target genes of the HH, NOTCH, and WNT pathways, and stem cell-related biomarkers (Table 2). As shown in Fig. 1 (representative scatter plots in Fig. S1), U–As was significantly associated with the expression of *GLI1* (β: — 0.11, 95% confidence interval [CI]: [— 0.21, 0.00]) and *POU5F1* (β: — 0.04, 95% CI: [— 0.09, 0.00]). A second, sequence-distinct Nanostring probe for *GLI1* (GLI1—2) also displayed a significant negative association with U–As (β: — 0.07, 95% CI: [— 0.12, — 0.01]). Based on the estimates from these multivariable linear regression models, *GLI1* and *POU5F1* levels would be expected to decrease 18% and 8%, respectively, between the 75th and 25th percentiles of U–As levels.

### 3.3 Placental Expression of Key Developmental Genes Associates Sex-specifically with Arsenic Exposure

**Fig. 2:**
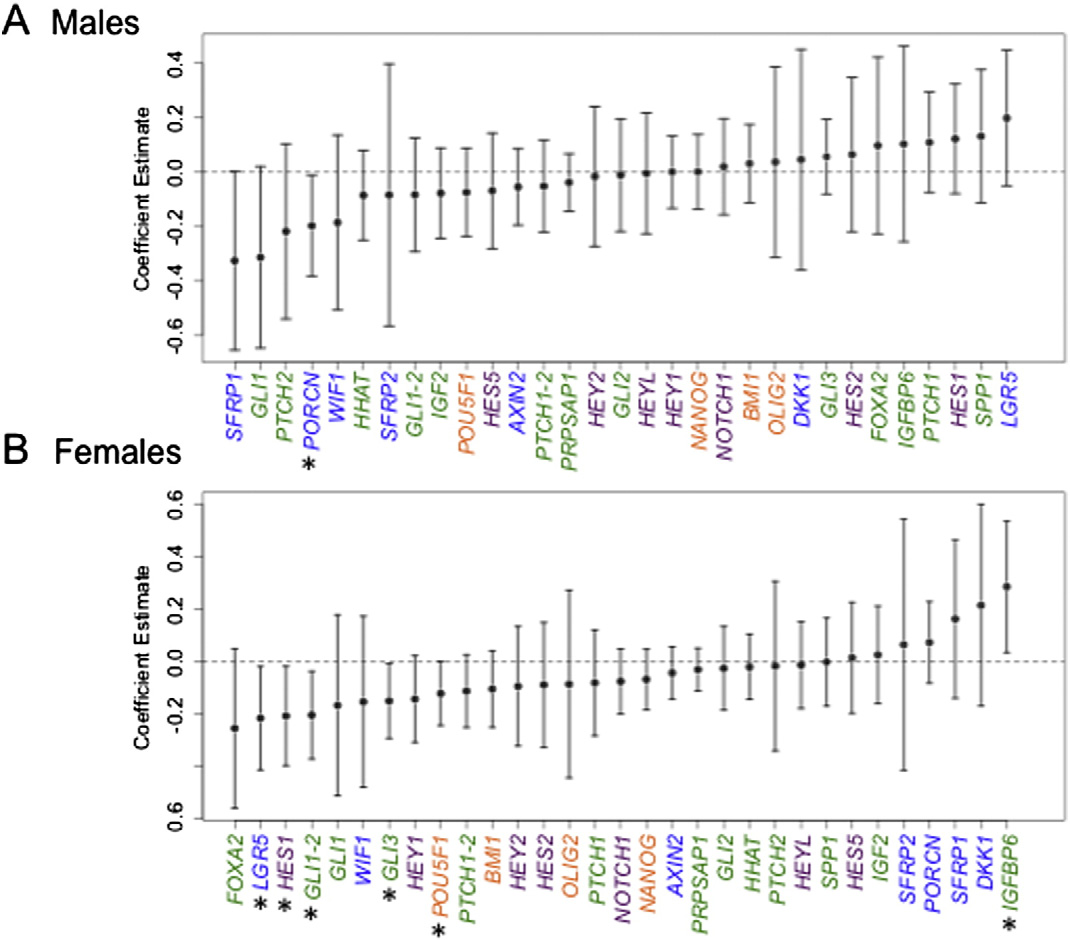
Sex-specific associations between placental gene expression and maternal U–As. Multivariable linear regression analyses were performed as in Fig. 1, after stratification of the cohort by infant sex, to determine the association between maternal U–As and placental gene expression in (A) male infants, and (B) female infants. The analyses were adjusted for maternal age. Dots depict coefficient estimates and error bars represent 95% CIs. Significant associations are those with 95% CIs not crossing zero (dotted line) and are marked by asterisks (*P < 0.05). Green; HH pathway-related genes, purple; NOTCH pathway-related genes, blue; WNT pathway-related genes, orange; stemness genes.

Next, as arsenic-related toxicities are reported to exhibit sexually dimorphic effects (Hamadani et al., 2011, Raqib et al., 2009, Xu et al., 2011), we analyzed the associations of U–As with our candidate genes after dividing the cohort into male and female subsets (Fig. 2). Interestingly, many more of the candidate genes showed associations with U–As among females than among males or the cohort as a whole. Specifically, in males, only one gene, *PORCN*, was associated with U–As (Fig. 2A; β: — 0.197, 95% CI: [— 0.383, — 0.011]), while in females, the expression of *LGR5*, *HES1*, *GLI1*, *GLI3* and *POU5F1* was negatively associated with U–As, and *IGFBP6* expression was positively associated (Fig. 2B; coefficient estimates and 95% CIs in Table S1). This observed sexual dimorphism was not due to differential arsenic exposure, as maternal U–As levels were not significantly different for male versus female infants (Fig. S2). Overall, this analysis indicates that a substantial number of key developmental genes are differentially affected by in utero arsenic exposure in male and female placentae. This suggests that there may be important sex differences in the impact of arsenic on fetal development, a notion that is in agreement with existing literature (Hamadani et al., 2011, Raqib et al., 2009, Xu et al., 2011).

### 3.4 Relationship between Maternal Arsenic Exposure and Infant Birth Weight

Previous studies have suggested that in utero arsenic exposure has negative effects on infant birth weight (Huyck et al., 2007, Llanos and Ronco, 2009, Rahman et al., 2009, Xu et al., 2011). In our cohort, we previously reported an expected weight loss of 1.30 g per 1-μg/L increase in U–As after adjustment for infant sex, maternal age and gestational age (p-value = 0.33) (Fei et al., 2013). Further stratification by infant sex revealed a decrease in birth weight of 0.15 g per 1-μg/L increase in U–As among males (p-value = 0.98), and a decrease in birth weight of 1.5 g per 1-μg/L increase in U–As among females (p-value = 0.21). While none of these associations reached statistical significance, probably due to insufficient sample size, the birth weight changes and trends in association with arsenic exposure in our cohort are similar in scale to what has been reported previously (Rahman et al., 2009, Hopenhayn et al., 2003).

### 3.5 Placental Expression of Key Developmental Genes Associates Sex-specifically with Infant Birth Weight

**Fig. 3:**
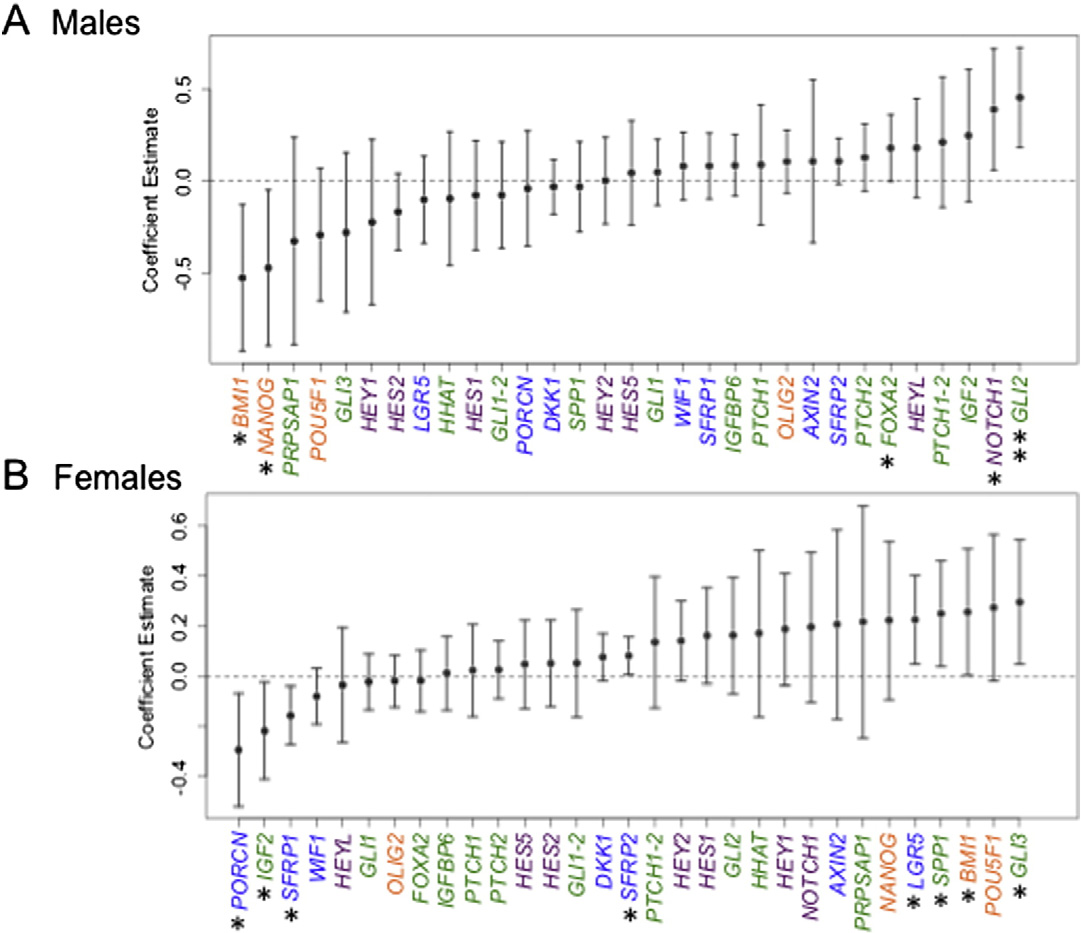
Sex-specific associations between placental gene expression and infant birth weight. Multivariable linear regression analyses were performed, after stratification of the cohort by infant sex, to determine the association between infant birth weight and placental gene expression for (A) male infants, and (B) female infants, after adjusting for maternal age at delivery and gestational age. Dots depict coefficient estimates and error bars represent 95% CIs. Significant associations are those with 95% CIs not crossing zero (dotted line) and are marked by asterisks (*P < 0.05, **P < 0.01). Green; HH pathway-related genes, purple; NOTCH pathway-related genes, blue; WNT pathway-related genes, orange; stemness genes.

We next examined whether the expression of our candidate genes was associated with birth weight, in order to assess their potential roles in mediating arsenic's effect on fetal growth. We performed multivariable linear regression analysis to examine our set of candidate genes for associations with infant birth weight (Fig. S3). In the cohort as a whole, birth weight was associated with the expression of three genes: *SFRP2*, encoding a WNT pathway antagonist (β: 0.090, 95% CI: [0.021, 0.160]); *NOTCH1*, encoding the NOTCH pathway receptor and target gene (β: 0.285, 95% CI: [0.066, 0.503]); and *GLI2*, encoding a HH pathway transcription factor (β: 0.301, 95% CI: [0.123, 0.480]). Similar to the associations of gene expression with U–As, more significant associations of gene expression with birth weight were found after stratifying the cohort by sex (Fig. 3). Among males, in addition to *GLI2* and *NOTCH1*, expression of the HH target gene *FOXA2* was positively associated with infant birth weight, while expression of two genes encoding stem cell regulators (*BMI1* and *NANOG*), was negatively associated with birth weight (Fig. 3A; coefficient estimates and 95% CIs in Table S2). Among females, expression of five genes (*SFRP2*, *LGR5*, *GLI3*, *SPP1* and *BMI1*) was positively associated with birth weight, while expression of three genes (*PORCN*, *IGF2* and *SFRP1*) was negatively associated with birth weight (Fig. 3B; coefficient estimates and 95% CIs in Table S3). These data show that the placental expression of several genes, encoding components of pivotal signaling pathways that drive early development, is associated with birth weight in a sexually dimorphic manner.

### 3.6 Expression of *GLI3* and *LGR5* in the Female Fetal Placenta is Associated with both Arsenic Exposure and Birth Weight

**Fig. 4:**
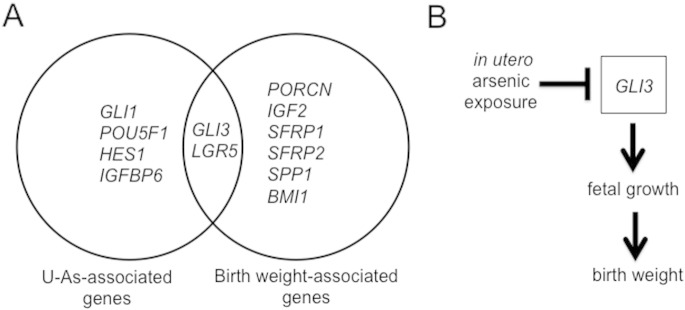
GLI3 as a potential mediator of birth weight regulation by arsenic in female placentae. (A) Venn diagram showing genes significantly associated with U–As and/or birth weight in female placentae (Figs. 2B, 3B). *LGR5* and *GLI3* are both negatively associated with U–As and positively associated with birth weight. (B) Model of GLI3 as an arsenic-responsive regulator of birth weight.

Finally, we compared the results of our sex-stratified multivariable linear regression analyses for U–As (Fig. 2) and birth weight (Fig. 3) to identify candidate genes that were significantly associated with both factors. In male placentae, no genes showed significant association of their expression with both U–As and birth weight. However, in female placentae, expression of *LGR5* and *GLI3* was negatively associated with U–As and positively associated with birth weight (Fig. 4A).

## 4 Discussion

In this study, we identified several key developmental and stem cell regulatory genes as potential biomarkers of arsenic exposure in the fetal placenta. Our results also revealed extensive sexual dimorphism in the associations between placental gene expression and both in utero arsenic exposure and infant birth weight. Particularly, we found that expression of the HH pathway component, *GLI3*, in female placentae was associated with both arsenic exposure and infant birth weight. Combined with our previous results implicating GLI3 repressor protein as a key arsenic target in cultured cells (Fei et al., 2010), these results suggest that GLI3 may be a pivotal signaling node affected by arsenic (Fig. 4B).

With respect to determining the molecular mechanisms underlying arsenic's effects on children's health, our study has some limitations that should be acknowledged. Firstly, this is a cross-sectional study, using single measurements of U–As at mid-gestation, and placental gene expression and infant weight at delivery. Therefore, this study was not designed to identify cause-and-effect relationships. Additionally, the chronological gap between the urinary arsenic measurement and analyses of birth weight and placental gene expression may have led to inaccurate assessment of arsenic exposure for some individuals, although previous studies show that an individual's urinary arsenic concentration is generally a reliable indicator of long-term exposure levels (Kile et al., 2009, Navas-Acien et al., 2009). Finally, our study was performed on a relatively small cohort and so was statistically underpowered to measure the direct relationship between arsenic exposure and birth weight, although the trend was similar to previous reports. Despite these limitations, this study highlights possible genes and pathways mediating arsenic's effects on fetal development and growth, providing an important basis for future studies to confirm the causative relationships.

Arsenic exposure levels in our population were relatively low and the gene expression changes we observed were also relatively subtle. Nonetheless, even small variations in the expression of key developmental pathway genes in utero could still impact fetal development and growth by influencing placental function. Moreover, since arsenic has been shown to readily pass across the placenta (Concha et al., 1998), it is likely that the gene expression changes we observed in the fetal placenta also occur in other fetal tissues, and directly influence their development and growth. In our cohort, we observed arsenic-associated reductions in the expression of the HH pathway target *GLI1*, and the stem cell regulator, *POU5F1*. POU5F1 (also called OCT4) is a critical player in maintaining pluripotency of embryonic stem cells, and its down-regulation is necessary for differentiation of the trophectoderm, which ultimately forms the placenta (Nichols et al., 1998). Interestingly, residual expression of *POU5F1* is detected throughout the first trimester and in term placenta tissues, and reduction in this residual expression is associated with gestational trophoblastic diseases (Zhang et al., 2008). In the fetus, early loss of *POU5F1* expression leads to preimplantation lethality (Nichols et al., 1998), and during later development, POU5F1 is essential for convergent extension and primordial germ cell survival (Kehler et al., 2004, Deveale et al., 2013). The loss of one copy of this gene, causing a reduction in expression of 30–40%, can induce differentiation of embryonic stem cells in vitro (Nichols et al., 1998). Thus, an 8% reduction in *POU5F1* expression, which we observe in response to arsenic exposure between the 25th and 75th quartiles of U–As levels, may have multiple effects on fetal health.

Relatively little is known about the role of GLI1 in placenta biology. However, in mice, several components of the HH pathway, including all three HH ligands; SMO; PTCH1; and the transcription factors GLI2 and GLI3, are expressed abundantly in fetal placenta (Pan et al., 2015), and disruption of HH activity causes severe placental defects, suggesting essential roles in normal placental development and possibly pregnancy maintenance (Pan et al., 2015, Jiang and Herman, 2006). *Gli1* homozygous null mice have reduced body weight, increased postnatal lethality, and defects in T-cell development, while heterozygotes are of normal weight but have reduced bone mass, reflecting the importance of normal levels of GLI1 for bone homeostasis and immune function (Kitaura et al., 2014, Drakopoulou et al., 2010). Moreover, a common single nucleotide polymorphism (SNP) in human *GLI1*, which reduces protein activity by around 50%, is associated, in both homozygous and heterozygous states, with increased risk of inflammatory bowel disease (Lees et al., 2008). Therefore, subtle changes in *GLI1* expression, such as the 18% reduction we observe in response to arsenic exposure between the 25th and 75th quartiles of U–As levels, may have important consequences for the fetus.

Interestingly, when we stratified our cohort by infant sex, more genes showed an association with arsenic exposure, and many of these associations were sexually dimorphic. In keeping with these results, previous studies have suggested that in utero arsenic exposure has differential effects on male and female infants. A study of a Chinese population identified a negative relationship between maternal arsenic exposure and birth weight among male, but not female infants (Xu et al., 2011). Differences in susceptibility to infections have also been noted, i.e. association of arsenic exposure with acute respiratory infections in male but not female children (Raqib et al., 2009). In addition, a neurobehavioral study of children in Bangladesh found that the adverse effects of in utero arsenic exposure on various IQ measures were sex-specific (Hamadani et al., 2011). We also observed considerable sexual dimorphism in the genes associated with birth weight between males and females, suggesting that the primary genes and pathways influencing birth weight may differ between the sexes, as is supported by recent studies in both mouse and human (Delahaye et al., 2014, Miranda et al., 2014). Of note, the *IGF2*, *PORCN*, *SPP1*, and *GLI3* genes, which are associated with birth weight among female placentae in our cohort, have been previously linked to birth weight variation in humans or animal models (Liu et al., 2012, Demetriou et al., 2014, Allan et al., 2007, Huang et al., 2013, Martin et al., 2007). Our findings suggest that infant sex is an important modifying factor in examining gene regulation during development.

Among our candidate genes, we identified *GLI3* as a potential mediator of birth weight regulation by arsenic, being both negatively associated with U–As and positively associated with birth weight in female infants. *GLI3* encodes a transcription factor that can act either as a transcriptional activator or repressor depending on its proteolytic status. We observed an arsenic-associated reduction in levels of both *GLI3* and the HH target *GLI1* in female placentae, suggesting that GLI3 acts primarily as an activator of HH signaling in this tissue. Heterozygous mutations in *GLI3* lead to congenital disorders including Greig cephalopolysyndactyly and Pallister–Hall syndrome (Naruse et al., 2010), characterized by polydactyly, syndactyly, and head defects. The Jackson extra-toes (X^tJ^) mouse, which expresses a non-functional splice variant of GLI3, has similar defects (Buscher et al., 1998, Maynard et al., 2002), and interestingly, heterozygous X^tJ^ mice have increased birth weight compared to wild-type mice (Martin et al., 2007). In the mouse placenta, knockdown of GLI3 partially rescues abnormal branching of the placental labyrinth caused by SHH knockout (Pan et al., 2015), suggesting that GLI3 controls placental morphogenesis by repressing SHH targets. While these mouse studies suggest that GLI3 negatively regulates HH signaling in the placenta and fetal growth, which contrasts with the positive regulation suggested by our findings, this may be explained by sex- or species-specific differences or compensatory mechanisms. In addition, a recent study in cattle identified two synonymous SNPs in *GLI3* that were associated with increased birth weight in both homozygous and heterozygous states (Huang et al., 2013). Together, these studies raise the possibility of a conserved role for *GLI3* in fetal growth regulation, and suggest that even subtle changes in its expression may impact birth weight. We previously showed that chronic exposure of NIH3T3 cells to arsenic reduced levels of GLI3 repressor protein and concomitantly increased HH pathway activity (Fei et al., 2010). Together with our current findings, this suggests that modulation of *GLI3*, both at the transcriptional and post-transcriptional levels, provides an important means by which arsenic affects various human pathologies.

## Author Contributions

D.L.F., A.J.C., E.L., M.R.K. and D.J.R. designed research; D.L.F. and C.G. performed gene expression profiling in placenta; D.C.K. and E.W. analyzed data; D.L.F., D.C.K., E.F.W., M.R.K. and D.J.R. wrote the paper.

## Competing Interests

The authors declare no competing interests.

## Figures and Tables

**Table 1:** Study cohort demographic information.

Characteristic	Mean (SD)	Number (%)	Median (interquartile range)
Number of pregnant women		133	–
Gestational age (wks)	39.5 (1.6)	–	–
Maternal age at enrollment (yrs)	31.1 (4.6)	–	–
Parity	1.1 (1.1)	–	–
Pre-pregnancy BMI (kg/m^2^)	24.9 (4.7)	–	–
Smoking status during pregnancy			
Never	–	97 (72.9)	–
Former	–	13 (9.8)	–
Current	–	5 (3.8)	–
Unknown	–	18 (13.5)	–
Number of infants	–	133	–
Infant birth weight (kg)	3.4 (0.4)	–	–
Infant sex			
Male	–	65 (48.9)	–
Female	–	68 (51.1)	–
Household water arsenic (μg/L)	–	–	0.36 (0.02–3.55)
Total urinary arsenic (U–As, μg/L)	–	–	4.4 (1.8–11.9)

**Table 2:**
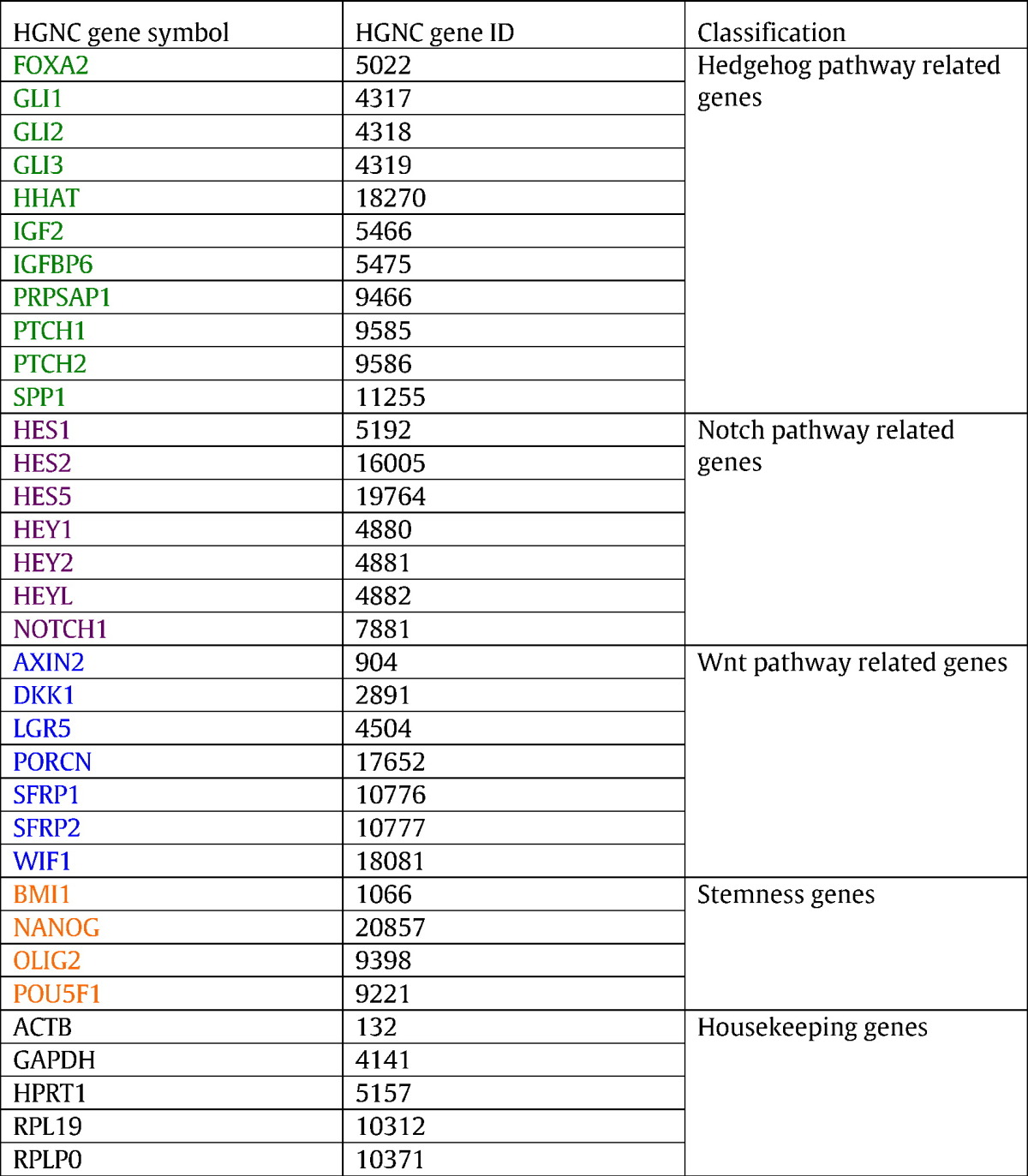
Candidate genes for association with in utero arsenic exposure and birth weight.

## Appendix A Supplementary data

Supplementary material Fig. S1. Association between U-As and the placental expression of *GLI1* and *PTCH1* (scatter plot views). (A) *GLI1*, (B) *PTCH1*. Fig. S2. Maternal U-As for male and female infants in the study cohort. Upper and lower ends of boxes indicate the 25th and 75th percentiles, respectively, and black band represents the median. Error bars represent minimum and maximum values, excluding outliers, which are depicted as open dots. P values are based on a Wilcoxon signed rank test. Fig. S3. Associations between placental gene expression and infant birth weight. Multivariable linear regression analyses were performed to determine the association between infant birth weight and placental gene expression, after adjusting for maternal age at delivery and gestational age. Dots depict coefficient estimates and error bars represent 95% CIs. Significant associations are those with 95% CIs not crossing zero (dotted line) and are marked by asterisks (* P <  0.05, ** P < 0.01). Green; HH pathway-related genes, purple; NOTCH pathway-related genes, blue; WNT pathway-related genes, orange; stemness genes. Table S1. Significant associations with U-As in females (Figure 2B). Table S2. Significant associations with birth weight in males (Figure 3A). Table S3. Significant associations with birth weight in females (Figure 3B). Table S4 (provided as separate Excel file). Unadjusted multivariable linear regression analyses of associations between placental candidate gene expression and maternal U-As or birth weight.
