# Supplementary material for: GLI3 Links Environmental Arsenic Exposure and Human Fetal Growth
Source: eBioMedicine. 2015 May 1;2(6):536–43. doi: 10.1016/j.ebiom.2015.04.019 (PMC4535308; doi:10.1016/j.ebiom.2015.04.019)
Supplement: Supplementary material — Fig. S1. Association between U-As and the placental expression of GLI1 and PTCH1 (scatter plot views). (A) GLI1, (B) PTCH1. Fig. S2. Maternal U-As for male and female infants in the study cohort. Upper and lower ends of boxes indicate the 25th and 75th percentiles, respectively, and black band represents the median. Error bars represent minimum and maximum values, excluding outliers, which are depicted as open dots. P values are based on a Wilcoxon signed rank test. Fig. S3. Associations between placental gene expression and infant birth weight. Multivariable linear regression analyses were performed to determine the association between infant birth weight and placental gene expression, after adjusting for maternal age at delivery and gestational age. Dots depict coefficient estimates and error bars represent 95% CIs. Significant associations are those with 95% CIs not crossing zero (dotted line) and are marked by asterisks (* P < 0.05, ** P < 0.01). Green; HH pathway-related genes, purple; NOTCH pathway-related genes, blue; WNT pathway-related genes, orange; stemness genes. Table S1. Significant associations with U-As in females (Figure 2B). Table S2. Significant associations with birth weight in males (Figure 3A). Table S3. Significant associations with birth weight in females (Figure 3B). Table S4 (provided as separate Excel file). Unadjusted multivariable linear regression analyses of associations between placental candidate gene expression and maternal U-As or birth weight. [file mmc1.zip › Supplemental Materials.docx]

**Supplemental Materials**

**Supplementary figures**


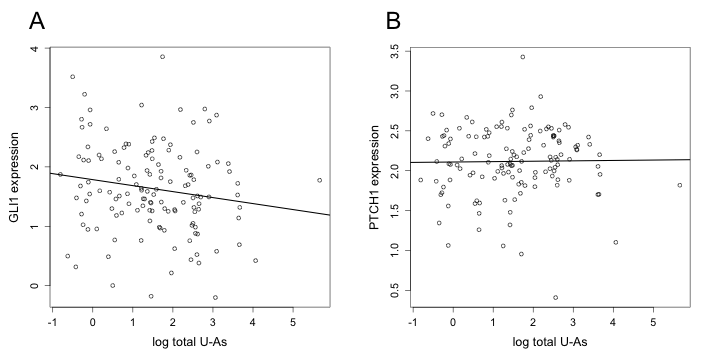


**Fig. S1.** Association between U-As and the placental expression of *GLI1* and *PTCH1* (scatter plot views). (A) *GLI1,* (B) *PTCH1*.


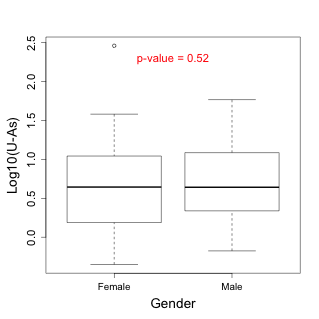


**Fig. S2.** Maternal U-As for male and female infants in the study cohort. Upper and lower ends of boxes indicate the 25th and 75th percentiles, respectively, and black band represents the median. Error bars represent minimum and maximum values, excluding outliers, which are depicted as open dots. P values are based on a Wilcoxon signed rank test.


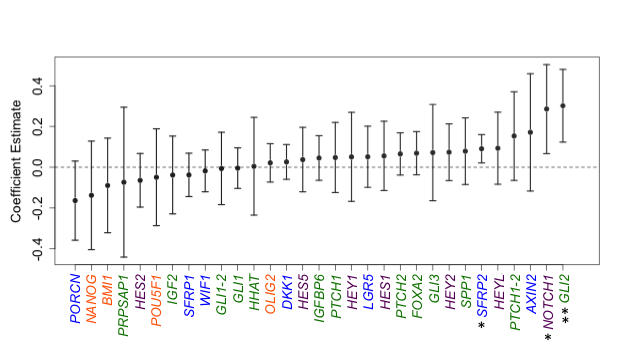


**Fig. S3.** Associations between placental gene expression and infant birth weight. Multivariable linear regression analyses were performed to determine the association between infant birth weight and placental gene expression, after adjusting for maternal age at delivery and gestational age. Dots depict coefficient estimates and error bars represent 95% CIs. Significant associations are those with 95% CIs not crossing zero (dotted line) and are marked by asterisks (* P < 0.05, ** P < 0.01). Green; HH pathway-related genes, purple; NOTCH pathway-related genes, blue; WNT pathway-related genes, orange; stemness genes.

**Supplementary tables**

| **Gene symbol** | **Coefficient estimate** | **Standard error** | **95% confidence interval** |
| --- | --- | --- | --- |
| LGR5 | -0.215 | 0.100 | [-0.412, -0.018] |
| HES1 | -0.207 | 0.097 | [-0.396, -0.018] |
| GLI1 | -0.166 | 0.176 | [-0.511, 0.178] |
| GLI3 | -0.150 | 0.073 | [-0.292, -0.008] |
| POU5F1 | -0.122 | 0.062 | [-0.243, 0.000] |
| IGFBP6 | 0.285 | 0.128 | [0.035, 0.536] |

**Table S1.** Significant associations with U-As in females (Figure 2B)

| **Gene symbol** | **Coefficient estimate** | **Standard error** | **95% confidence interval** |
| --- | --- | --- | --- |
| GLI2 | 0.455 | 0.139 | [0.182, 0.727] |
| NOTCH1 | 0.390 | 0.168 | [0.060, 0.720] |
| FOXA2 | 0.180 | 0.093 | [-0.003, 0.362] |
| BMI1 | -0.528 | 0.204 | [-0.927, -0.129] |
| NANOG | -0.474 | 0.217 | [-0.899, -0.049] |

**Table S2.** Significant associations with birth weight in males (Figure 3A)

| **Gene symbol** | **Coefficient estimate** | **Standard error** | **95% confidence interval** |
| --- | --- | --- | --- |
| SFRP2 | 0.080 | 0.038 | [0.005, 0.156] |
| LGR5 | 0.225 | 0.091 | [0.047, 0.403] |
| GLI3 | 0.295 | 0.127 | [0.047, 0.544] |
| SPP1 | 0.249 | 0.108 | [0.037, 0.461] |
| BMI1 | 0.255 | 0.129 | [0.003, 0.508] |
| PORCN | -0.294 | 0.116 | [-0.520, -0.067] |
| IGF2 | -0.218 | 0.099 | [-0.411, -0.025] |
| SFRP1 | -0.157 | 0.059 | [-0.273, -0.040] |

**Table S3.** Significant associations with birth weight in females (Figure 3B)

**Table S4 (provided as separate Excel file).** Unadjusted multivariable linear regression analyses of associations between placental candidate gene expression and maternal U-As or birth weight
